# West Nile Virus Encephalitis and Myocarditis in Wolf and Dog

**DOI:** 10.3201/eid0910.020617

**Published:** 2003-10

**Authors:** Carol A. Lichtensteiger, Kathleen Heinz-Taheny, Tanasa S. Osborne, Robert J. Novak, Beth A. Lewis, Margaret L. Firth

**Affiliations:** * University of Illinois, Champaign-Urbana, Illinois, USA; † Town and Country Animal Hospital, Normal, Illinois, USA

## Abstract

In the third season (2002) of the West Nile virus epidemic in the United States, two canids (wolf and dog) were diagnosed with West Nile virus encephalitis and myocarditis with similarities to known affected species (humans, horses, and birds). The West Nile virus infections were confirmed by immunohistochemistry and polymerase chain reaction.

Since its 1999 introduction in New York, West Nile virus (WNV) has spread to >40 states, causing seasonal mosquito-borne disease in humans, horses, and birds (*1*–*7*). We recently identified two Illinois canids (a captive wolf and a domestic dog) with severe disease associated with WNV infection.

Outside the Western Hemisphere, WNV has been endemic for decades (*4*,*8*–*11*). Canids have not been thought to be important in the epidemiology of this virus. However, a dog with neurologic disease that died in Africa in 1977 is now thought to have been infected with WNV (*9*,*12*). In a recent study in which four dogs were experimentally infected, no clinical disease was observed, and a low viremia developed in one dog (*11*). Natural infections occur in dogs, as indicated by serum antibodies; seropositivity in surveys was 37% in the 1980s in South Africa, 24% in the 1990s in India, and 5% in 1999 in New York City (*10*,*11*,*13*). In addition, a few individuals of some mammalian species have been listed as infected (not diseased): 14 bats, four rodents, three rabbits, two cats, two raccoons, and a skunk (*6*).

## The Study

Two Illinois canids were brought in for necropsy in August 2002: 1) a 3-month-old female wolf pup, which died after 2 days of lethargy, depression, and irritability that progressed to anorexia, weakness, ataxia, and blindness and 2) an overweight 8-year-old castrated male Irish Setter-Golden Retriever mixed breed dog that was euthanized in moribund condition after a 7-day illness. The dog was hospitalized after 3 days of lethargy, anorexia, polydipsia, ocular discharge, and difficulty in rising that had progressed to fever, listlessness, weakness, ptyalism, nasal and ocular discharge, watery diarrhea, and abdominal pain. Although the diarrhea resolved and the dog was stronger and more alert the morning after hospitalization, over the next 3 days the multisystemic clinical signs developed, including dyspnea, diarrhea with melena, ataxia, a head tilt with head bobbing, pulmonary edema, and cardiac arrhythmias. While hospitalized, the dog had mild anemia without spherocytes, moderate to severe thrombocytopenia with large platelets, moderate leukocytosis with a left shift, a moderate hypokalemia, a mild hypoglycemia, and hypoproteinemia. Symptomatic and supportive care treatment included broad-spectrum antibiotics, a gastrointestinal protectant, a diuretic, fluid therapy, and systemic corticosteroids (for immune-mediated thrombocytopenia and anemia).

Both the wolf and dog were necropsied. Samples of multiple tissues were fixed and processed for routine diagnostic histopathology. In addition, tissues were screened with peroxidase immunohistochemistry for distemper, rabies, WNV, *Toxoplasma,* and *Neospora*. Sections for WNV immunohistochemistry were pretreated with 0.1% protease (20 min at 37°C) and nonspecific antibody binding blocked with Power Block (BioGenx, San Ramon, CA). The primary antibody was mouse anti-WNV monoclonal ascitic fluid diluted 1:1,000 (ATTC, Manassas, VA). The wolf brain was assayed for rabies antigen by fluorescent antibody (Public Health Laboratory, Springfield, IL).

West Nile viral RNA in the brain of the wolf and the kidney and liver of the dog (available cyropreserved tissue) was assayed by using a modified 5′ nuclease fluorogenic real-time, reverse transcriptase polymerase chain reaction assay (RT-PCR) (TaqMan, Applied Biosystems, Foster City, CA) (*14*). Each amplification reaction contained primers and probes (Table) (*14*,*15*) for both WNV and St. Louis encephalitis virus (SLEV) with FAM (6-carboxyfluorescein phosphoramidite = 6-carboxyfluorescein) and VIC (Applied Biosystems) fluorescent-labeled probes, respectively. A tissue sample (about 200 µg) was homogenized in 1.5 mL of medium 199 with L-glutamine, Hanks balanced salt solution, and 25 mM HEPES in a microfuge tube with three 3.2-mm stainless steel beads (Biospec Products, Bartlesville, OK). The sample was ground in a mixer mill for four min at a rate of 1/30 sec^-1^, and then centrifuged (4,000 X *g,* 4 min). RNA was purified from 220 µL of the supernatant by using a viral RNA purification kit and the Biorobot 9604 (QIAGEN, Inc., Alameda, CA), eluting in a final volume of 85 µL. Real-time, RT-PCR was done in a 25-µL reaction with One-step RT-PCR Master Mix kit (Applied Biosystems) containing 25 pmol of each primer set, 3.25 pmol of each probe, and 10 µL of the RNA extract. The amplification reaction was run in an Applied Biosystems Sequencing Detection System 7000 programmed for 48.0°C for 30 min, 95°C for 10 min, and 40 cycles of 95°C for 15 sec and 60°C for 1 min. RNA samples from WNV isolate NY99 (*5*) and SLEV were run in parallel for positive controls for the amplification.

**Table:** Sequence (5´ to 3´) of primers and probes used in real-time reverse transcriptase–polymerase chain reaction to assay for West Nile virus genome in canid tissue

Item	West Nile virus	St. Louis encephalitis virus
Forward primer	CAGACCACGCTACGGCG	GAAAACTGGGTTCTGCGCA
Reverse primer	CTAGGGCCGCGTGGG	GTTGCTGCCTAGCATCCATCC
Probe	TCTGCGGAGAGTGCAGTCTGCGAT	TGGATATGCCCTAGTTGCGCTGGC

**Figure 1:**
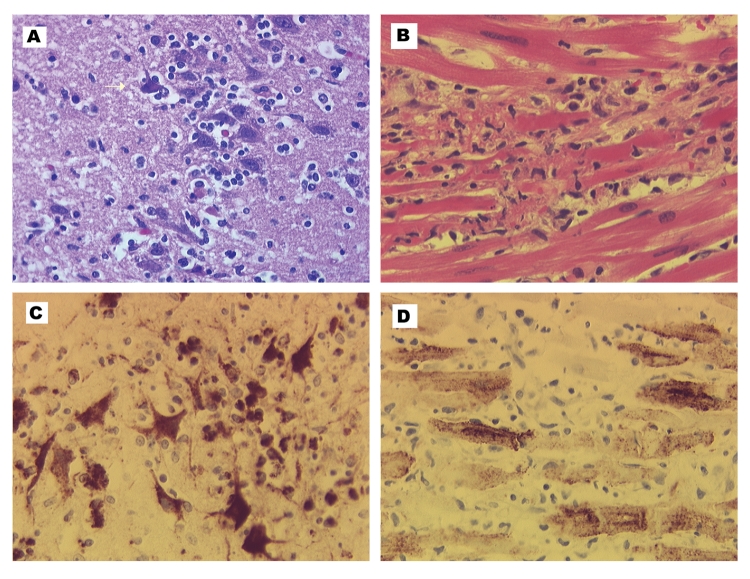
Encephalitis and myocarditis in two West Nile virus–infected canids. A, histopathology of the cerebrum of the wolf. Focus of lymphocyte infiltration and necrosis with mild gliosis; arrow indicates necrotic neuron. Hematoxylin-eosin staining. B, histopathology of the heart of the dog. Focus of leukocytic infiltration, mostly lymphocytes, and myocardial cell degeneration and necrosis. Hematoxylin-eosin staining. C, West Nile virus immunohistochemistry of the cerebrum of the wolf. Intense labeling of neurons in focus of inflammation. Some labeling is also in the glial cells or lymphocytes. Immunoperoxidase with hematoxylin counterstain. D, West Nile virus immunohistochemistry of the heart of dog. Intense labeling of most myocardial cells in the field. Immunoimmunoperoxidase with hematoxylin counterstain.

At necropsy, the wolf had no gross lesions. Histologically, the brain had encephalitis compatible with a viral infection, including scattered blood vessels with narrow rims of lymphocytes. Scattered in the gray matter of all brain sections were random, poorly demarcated aggregates of microglial cells and lymphocytes with rare neutrophils and mild necrosis including an occasional necrotic neuron (Figure 1A). The white matter contained rare glial nodules. Each of two sections of heart had a small focus of myocardial cell fragmentation with a few lymphocytes and macrophages and fewer neutrophils. A similar smaller focus was in one of the two sections of skeletal muscle. The outer cortex of the adrenal gland had several foci of two to five necrotic cells with a few lymphocytes. No major histologic changes were evident in the kidney, lung, liver, spleen, tonsil, pancreas, small intestine, colon, sciatic nerve, or bone marrow.

The dog had several abnormalities at necropsy. The major gross lesion was fibrinous epicarditis of the atria. Other important findings included a hepatopathy (mottled tan yellow with an uneven surface), pulmonary edema, and two acute splenic infarcts. Histologically, the dog had a polioencephalitis similar to the wolf’s, but much milder. In addition, the basal ganglia area had a focus of malacia predominately cleared via foamy macrophages. The dog had marked myocarditis (especially the atria) with numerous loose aggregates of leukocytes associated with degenerated or necrotic myocardial cells (Figure 1B). Lymphocytes predominated in the infiltrate; an occasional macrophage and neutrophil contributed. Both the lung and liver had changes compatible with heart failure: pulmonary edema, marked hepatic congestion, and marked hepatocellular fatty change with cholestasis. In addition to the acute infarcts, the spleen had marked lymphoid atrophy and marked hematopoiesis with hemosiderosis. The bone marrow was hyperplastic with proliferation of all three hematopoietic lineages. The hemogram, melena, and necropsy hematopoietic findings are most likely due to immune-mediated thrombocytopenia and anemia, a sporadic idiopathic condition in dogs.

No antigen was detected in either canid for canine distemper, rabies, toxoplasmosis, or neosporosis, diseases that can cause encephalitis or myocarditis. In contrast, WNV immunolabeling was intense in the brain of the wolf (Figure 1C). Abundant, intense labeling was associated with the areas of inflammation; focally, the labeling was grossly visible. Many neurons had intense labeling, and less dense labeling was associated with glial cells or lymphocytes. In the gray matter between inflammatory nodules, scattered individual neurons labeled intensely. Also immunolabeled in the wolf were a few myocardial cells and several small clusters of cells in the zona glomerulosa of the adrenal gland. No labeling was identified in the kidney and spleen. In the dog, the heart had intense immunolabeling in many myocardial cells (Figure 1D); the most extensive labeling was in the atria. The labeling in the dog brain was weak and inconclusive, and no label was found in the spleen. The dog had viral immunolabeling in scattered small foci of cells in the adrenal zona glomerulosa and renal tubular epithelial cells.

**Figure 2:**
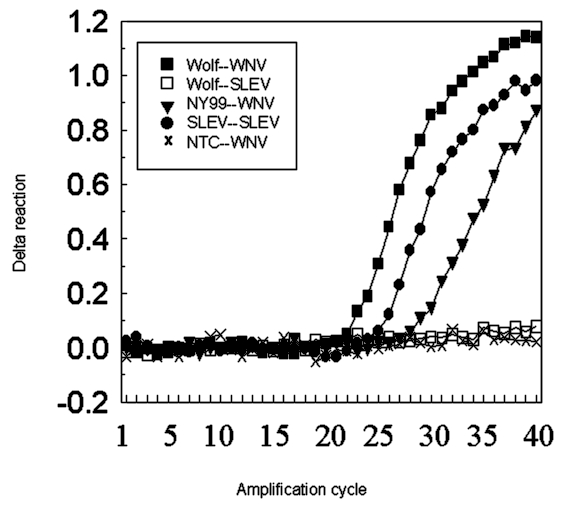
Real-time reverse transcriptase polymerase chain reaction of RNA extracted from the wolf brain. The amplification was duplexed with primers and probes for West Nile and St. Louis encephalitis viruses detected with fluorochrome dyes, FAM and VIC, respectively. Test and control samples were run in parallel and in duplicate (extraction and amplification) with consistent results. Delta reaction on the y axis represents the change in threshold fluorescence. The box lists the source of the template nucleic acid and the specificity of the fluorochrome probe (template source-probe specificity). WNV, West Nile virus; NY99, an isolate from the 1999 outbreak in New York; SLEV, St. Louis encephalitis virus; NTC, no template control.

In the PCR assay, the WNV amplicon was abundant in the samples from the wolf brain (Figure 2), the dog kidney (not shown), and NY99 isolate (Figure 2). Samples from the dog liver failed to generate an amplicon signal. The SLEV primers, as expected, generated amplicons for SLEV control genome; the primers did not generate amplicons from samples of canid tissue (Figure 2).

## Discussion

The dog and wolf reported here are the first canids reported with WNV disease in the U.S. outbreak. The encephalitis in the canids affected the gray matter similar to WNV disease in horses, whereas humans have inflammatory nodules in both the gray and white matter (*2*,*16*,*17*). The myocarditis in the dog more closely resembled the lesions of WNV-infected birds than the lesions reported in mammals (*2*,*3*,*17*). Although crows and blue jays usually die without evidence of inflammation, WNV antigen is frequently demonstrated by immunohistochemistry in the hearts and in infected raptors, myocarditis is a common finding (J. J. Andrews, pers. comm.). The dog apparently had a concurrent, immune-mediated disease, which may have increased susceptibility for WNV disease.

Both of the canids likely were infected by bites from infected mosquitoes. WNV has been detected in 12 species of mosquitoes in Illinois, and four of these species prefer mammalian hosts (R. Novak, unpub. data). Novel routes of infection are also possible, such as ingestion of infected birds. The epidemiology of WNV in canids is likely similar to that in humans: sporadic disease cases with no important role in viral transmission or maintenance. More investigation is needed to confirm the epidemiology of WNV in canids and to monitor disease in other known and novel host species.
